# Plant-Based Diets: Considerations for Environmental Impact, Protein Quality, and Exercise Performance

**DOI:** 10.3390/nu10121841

**Published:** 2018-12-01

**Authors:** Heidi Lynch, Carol Johnston, Christopher Wharton

**Affiliations:** 1 Point Loma Nazarene University, San Diego, CA 92106, USA; 2 Arizona State University, Phoenix, AZ 85004, USA; carol.johnston@asu.edu (C.J.); christopher.wharton@asu.edu (C.W.)

**Keywords:** vegetarian, vegan, plant-based, sustainability, health, nutrition, diet, athlete, exercise, protein

## Abstract

Plant-based diets provide well-established physical and environmental health benefits. These benefits stem in part from the degree of restriction of animal-derived foods. Historically, meat and other animal-derived proteins have been viewed as an integral component of athletes’ diets, leading some to question the adequacy of vegetarian or vegan diets for supporting athletic performance. The purpose of this review is to examine the impact of plant-based diets on human physical health, environmental sustainability, and exercise performance capacity. Based on currently available literature, it is unlikely that plant-based diets provide advantages, but do not suffer from disadvantages, compared to omnivorous diets for strength, anaerobic, or aerobic exercise performance. However, plant-based diets typically reduce the risk of developing numerous chronic diseases over the lifespan and require fewer natural resources for production compared to meat-containing diets. As such, plant-based diets appear to be viable options for adequately supporting athletic performance while concurrently contributing to overall physical and environmental health. Given the sparse literature comparing omnivore, vegetarian, and vegan athletes, particularly at the elite level, further research is warranted to ascertain differences that might appear at the highest levels of training and athletic performance.

## 1. Introduction 

Plant-based diets (including generally less animal-food intensive, vegetarian, or vegan diets) represent a growing area of interest in the promotion of physical and environmental health [1,2]. Reductions in risk for developing chronic diseases are linked to adherence to plant-based diets [3,4,5,6,7,8,9,10,11,12,13,14,15,16,17,18,19,20,21,22,23,24,25], and production of plant foods tends to be less resource-intensive and environmentally destructive for a number of reasons, especially due to lower levels of greenhouse gas emissions (GHGEs) compared to raising animals for human consumption [26,27,28,29,30,31,32,33,34,35,36,37,38,39,40]. However, in spite of well-documented human and environmental benefits of plant-based diets, among the general population, some continue to question the adequacy of plant-based diets in supporting exercise performance. This review addresses these issues by examining literature on differences between plant-based and meat-containing diets with respect to nutrient composition, human health, performance, and environmental impact. Particular focus is placed on differences between plant and animal proteins as well as discussion of literature comparing vegetarian, vegan, and omnivorous diets on exercise performance.

## 2. Vegetarian Diets and Health

Observational data show that vegetarians tend to have better cardiovascular outcomes compared to those consuming omnivorous diets, including a reduced risk of morbidity and mortality from ischemic heart disease [23,41,42]; reduced incidence of cancers, particularly among vegans [42]; decreased risk of developing Type 2 Diabetes [24,43]; decreased risk of developing metabolic syndrome (MetS) [44,45]; and lower all-cause mortality [5,37]. These positive health outcomes likely relate to the lower body mass index (BMI) [46]; lower glucose levels [42]; lower systolic and diastolic blood pressure [10,22]; lower total and low-density lipoprotein cholesterol [17,25]; lower triglycerides [16]; lower levels of uric acid and high-sensitivity C-reactive protein; and higher levels of plasma ascorbic acid observed among vegetarians [23]. Experimental studies utilizing vegetarian and vegan diets offer similar results. Dietary interventions in which participants adopt a vegetarian diet have demonstrated improvements in lipid profiles of participants including decreases in total cholesterol (TC), low-density lipoprotein cholesterol (LDL-C), and triglycerides (TG) [11,47]. Other research has shown reduced visceral fat, improved oxidative stress markers, and insulin sensitivity among patients with diabetes [20]. Although interventions utilizing vegan diets are less common, one experimental study resulted in a reduction in C-reactive protein (CRP), a marker of inflammation, over the course of a 3-week vegan dietary intervention [48]. Another 4-week vegan dietary intervention significantly reduced total medication use among participants due to reduced systolic and diastolic blood pressure and lipids [49]. 

Dietary choices affect the environment as well as human health. Producing plant protein generally requires less land [29,30,33,34], water [30,32,33,34], and energy [27,29,32,33,34] compared to producing animal protein and results in less GHGEs in aggregate [34,37,38,50,51]. Meat and other animal products require more life cycle inputs per kilogram (kg) of product than plant products as well [52]. Consequently, following a more plant-based diet is often considered the most effective strategy for systemically reducing GHGEs and agricultural land use related to food production and consumption [53]. One other environmental concern that is less well publicized, but certainly not less important, is the rapidly depleting global supply of high-quality, mineable phosphorus. Used as part of fertilizers for food production, known reserves of phosphorus are limited and could potentially be depleted within 50–100 years if consumption trends continue at the same rate [28,54]. Vegetarian diets require considerably less phosphorus to produce than meat-containing diets; as such, shifting broad-scale dietary patterns to less meat consumption could be a vital strategy to contend with a looming mineable phosphorus shortage [28].

Not all meat or animal protein exerts the same environmental toll, however. For example, switching from consuming ruminant meat (such as beef) to monogastric meat (such as poultry or pork) reduces GHGEs associated with the diet [55]. Avoiding overconsumption of nutrients, including protein (particularly animal protein), has been suggested as a means of reducing the environmental impact of a diet [56]. Based on life cycle assessment studies, 1 kg of protein from beef generated 45–640 kg of carbon dioxide equivalents (CO2e) in comparison to 10 kg CO2e per kg of protein from tofu [57]. Notably, pork, chicken, and seafood fared better than beef with ranges of 20–55, 10–30, and 4–540 kg CO2e per kg of protein, respectively [58]. Although vegan and vegetarian diets dramatically reduce GHGEs, following a healthy, yet less-animal intensive diet compared to the average American diet would also provide environmental benefits [55,59]. Wirsenius et al. (2010) calculated that a 25% reduction in meat consumption and transition to vegetarian eating patterns would minimize the impact of agricultural land expansion on ecosystems, biodiversity, and carbon dioxide emissions [60]. Other researchers call for greater reductions in animal protein consumption. For example, Machovina and colleagues (2015) encourage a global reduction in animal product consumption to less than 10% of total calories, an amount approximately equivalent to 100 g of animal product, or the amount of meat about the size of a deck of playing cards. This goal is predicated upon balancing the needs of nutritional health, ecological footprint, and people’s desires to eat meat [53]. Others have urged per capita global reductions down to 90 g of animal products per day [61]. Another option for high-quality protein that is less-resource intensive compared to traditional animal-based protein is edible insects. On average, the protein content of edible insects meets or exceeds the indispensable amino acid requirements for adults and is comparable to that of beef, eggs, milk, and soy [62]. Various types of insects have been part of traditional diets in tropical and sub-Saharan societies, and references to consuming insects in the United States are documented as early as 1885 [63]. One benefit of consuming insects for protein is their high feed conversion efficiency. To calculate an animal or insect’s feed conversion ratio (FCR), one divides the mass of the feed consumed by the mass of the edible component [64]. FCRs for insects have been reported to range from 1.47 to 5.3 [64], whereas they range from 6–25 for beef [65]. Additionally, edible insects require little land and emit low levels of greenhouse gas emissions compared to traditional animal husbandry [63]. As of the time of writing this paper, 584 products are available for purchase on Amazon.com using the search phrase “insect protein,” including cricket protein powder, cricket flour, whole “gourmet” crickets, and cricket protein bars [66]. Inclusion of insect protein has recently been encouraged for athletes as well [67].

The choice of whether to include animal-derived protein (dairy, eggs, meat, fish, or poultry) in the diet may relate to concerns about physical health, environmental sustainability, ethics related to worker or animal welfare, or religious convictions among other motivations. Consumer interest in plant-based nutrition is growing, as evidenced by the increase in Google searches for the term “vegan” over the past 5 years [68]; 2016 being named the “International Year of Pulses” by the United Nations [69]; and Mintel predicting that annual food trends will see a continued emphasis on sustainability, vegetarian, vegan, and other plant-focused food formulations [1]. Additionally, in 2018, Mintel reported that “natural” and “ethical” claims were rising for food and drink products, and that animal-free “dairy” and laboratory-grown meat were being increasingly pursued in development to help address the problem of resource-intensive animal agriculture. Mintel predicts that such scientifically engineered food will become more acceptable to the mainstream population due to growing eco-consciousness [2]. The type of animal protein being consumed is shifting, too, according to a report by the United States Department of Agriculture Economic Research Service (USDA ERS), indicating that consumption of red meat is steadily decreasing whereas consumption of poultry is increasing [70].This shift in types of animal protein being consumed likely relates in part to growing eco-consciousness and to increased understanding of the association between red meat consumption and increased risk of cancer and other chronic diseases [71].

## 3. Protein Quality and Current Recommendations

Several features differentiate plant-based protein from animal-based protein, in addition to the source. Of the indispensable amino acids, branched-chain amino acids (BCAAs) are particularly important for promoting muscle protein synthesis (MPS) and include leucine, isoleucine, and valine. These amino acids are more concentrated in animal-based protein compared to plant protein [72]. Digestion and absorption rates of different proteins can also differ and thus impact postprandial MPS rates. As such, some proteins (such as whey) are considered “fast” since they are rapidly digested, resulting in amino acids appearing quickly in the bloodstream. Other proteins, such as casein, are considered “slow” since they result in a slower, more prolonged, rate of absorption [73]. Soy protein is also considered a fast protein, although it does not stimulate MPS to the same extent as whey protein, even when both protein sources provided 10 g of essential amino acids, an amount generally considered sufficient to induce maximal MPS in young adults (about age 30) [74,75]. This may be due to differences in amino acid composition (specifically, a lower leucine content) [75]. Since it has been suggested that total leucine content may be of primary importance in driving muscle protein anabolic responses [76], more research is needed to investigate MPS in response to protein doses matched for total leucine content. Interestingly, intervention studies utilizing either a whey protein or soy protein supplement in conjunction with strength training, typically yield negligible differences between groups for lean mass development [77,78,79]. Another difference between animal or plant proteins is the presence or absence of antinutritional factors, compounds that affect digestibility of the protein. Antinutritional factors naturally occur more often in vegetable foods and include glucosinolates, trypsin inhibitors, hemagglutinins, tannins, phytates, and gossypol. They may also be formed by heat or alkaline processing of animal and vegetable protein foods [80]. 

These factors work in different ways to reduce the digestibility of protein, and they are often found in plant foods such as beans, legumes, soybeans, and cereals. One mechanism of action includes disruption of enzymes involved in protein digestion. For example, trypsin, a pancreatic enzyme, is involved with breaking down protein. Trypsin inhibitors in raw soybean meal, beans, legumes, cereals, tomatoes, and potatoes result in less efficient protein digestion [81]. Tannins, found in sourghum, millet, barley, legume seeds, beans, and peas, reduce the digestibility of carbohydrates, minerals, and protein, likely by inhibiting digestive enzymes. Tannins further influence digestion by chelating certain mineral cofactors of digestive enzymes, thereby reducing enzymatic action [82]. Phytate, found in nuts, seeds, and grains, binds with proteins in the digestive tract, reducing their absorption [83]. However, the impact of antinutritional factors can also be lessened through a variety of preparation techniques. Soaking, fermentation, and germination all can reduce levels of phytate in foods [84]. In addition, home processing or commercial canning of beans improves the digestibility of protein, with home cooking resulting in better digestibility compared to commercial preparation [80]. This is likely due to the reduced formation of complexes between trypsin inhibitors and trypsin, thus freeing more of the enzymes for protein digestion. For a more comprehensive review of antinutritional factors and their mechanisms of action, readers are directed to a review by Gilani and colleagues (2005) [80].

Despite the more frequent occurrence of antinutritional factors in vegetable-based protein foods, consuming a balanced vegetarian or vegan diet that includes a variety of plant protein sources has consistently been shown to be nutritionally adequate in terms of providing sufficient amounts of essential amino acids [85,86]. As such, these diets are still supported and promoted by major food and nutrition organizations such as the Academy of Nutrition and Dietetics [86,87,88]. 

In order to compare protein foods effectively in relation to amino acid composition, digestibility, and overall quality, various scoring systems for protein-containing foods have been created. One of the most widely used methods was developed in 1989 when the Food and Agriculture Organization and the World Health Organization convened an expert consultation to quantify the quality of proteins through the Protein Digestibility Corrected Amino Acid Score (PDCAAS) [89]. While the PDCAAS has been helpful for comparing protein quality between foods, it has suffered limitations. First, PDCAAS scores are truncated at 1.00, even though some proteins could have higher values than 1.00 if this were permitted by the method [89,90]. Second, PCDAAS values likely overestimate protein quality since the method relies upon fecal (not illeal) analysis of protein digestibility. This is problematic since some of the nitrogen disappearance in the large intestine is due not to protein digestion and absorption, but rather to microbial degradation, resulting in ammonia production, absorption, and excretion as urine [91]. Given these concerns, an updated assessment of protein quality was created in 2011 by the Food and Agriculture Organization Expert Consultation. This newer system is called the Digestible Indispensable Amino Acid Score (DIAAS) [90]. DIAAS is considered a superior measure of protein quality because it is calculated using illeal digestibility, and values are not truncated at 1.0, as they are with the PDCAAS. Typically, animal proteins have higher PDCAAS and DIAAS scores compared to plant proteins, reflecting the higher digestibility. 

In spite of these protein quality assessments, current recommendations regarding the amount of protein to consume do not directly address the quality of protein. The Acceptable Macronutrient Distribution Range (AMDR), created by the Food and Nutrition Board of the Institute of Medicine (now the Health and Medicine division of the National Academies of Science), suggests a range of protein intake providing between 10–35% of daily calories in the diet, and the Recommended Dietary Allowance (RDA) for protein is 0.80 g of “good quality protein” per kg of body weight per day (g/kg/day) [92]. These recommendations were originally developed based on the minimum amount of protein needed to achieve body nitrogen equilibrium (zero balance) in healthy adults as determined by nitrogen balance studies [93]. However, results derived from improved methodologies, such as the indicator amino acid oxidation technique, suggest higher protein intakes (1.2–1.6 g/kg/day) to optimize health above and beyond simply meeting minimal needs for the general population [94]. The concept of optimizing health versus meeting minimal needs for various nutrients is nuanced and remains open to debate as the definition of “optimal health” may vary depending upon one’s goals (for example, preventing chronic disease, enhancing MPS for building muscle maintenance to help prevent sarcopenia in older age, or maximizing MPS in relation to training and performance outcomes). 

Variables that affect dietary protein requirements to optimize MPS include age, physical activity expenditure, and energy balance. Young adults need less protein per meal or snack (0.24 g/kg) to maximally stimulate MPS compared to older adults (0.40 g/kg) [95]. Physical activity level also affects protein needs. Although the Dietary Reference Intakes do not make specific provisions for athletes, the American College of Sports Medicine, the Academy of Nutrition and Dietetics, and Dietitians of Canada in a joint position statement recommended athletes consume 1.2–1.7 g/kg/day [85]. During times of energy restriction, in order to promote retention of lean body mass, athletes may be encouraged to consume up to 2.0 g/kg/day [96]. 

Timing and distribution of protein consumption throughout the day are also important factors impacting MPS. It is not adequate to focus solely on the total amount of protein per day without considering the distribution and timing of protein intake. Consuming protein evenly throughout the day instead of in skewed distributions results in greater MPS [68], which is advantageous for building and preserving lean body mass. Additionally, exercising prior to consuming protein enhances anabolic responsiveness to dietary protein consumption [97,98]. Distributing protein consumption throughout the day may include a pre-sleep protein feeding. Recent work has shown that this promotes MPS overnight, a time when people are typically not in an anabolic state, and the response is augmented when preceded by resistance training [99].

## 4. Nutrient Intake and Diet Quality by Dietary Pattern

In spite of the marked differences in food consumption of vegetarians, vegans, and omnivores, literature about the effect of dietary patterns on athletic or exercise performance outcomes is sparse. Useful literature in this area, however, might come from observational studies. Although these studies cannot offer the insights that randomized controlled trials (RCTs) can, observational designs might still be advantageous for comparing vegetarian and omnivorous athletes. Many exercise or sport-related RCTs assign omnivores to a vegetarian diet for brief periods of time, yet extrapolate conclusions to athletes who follow vegetarian diets long-term. It is likely that brief interventions do not reflect completely the effect on athletic performance of plant-based dietary patterns maintained for longer durations. There are broad-scale differences in nutrient intake and nutrient profiles among vegans, vegetarians, and omnivores, hence, the length of adherence to a dietary pattern may also factor into exercise and fitness-related outcomes including cardiorespiratory fitness, strength, and power. 

Nutrient intakes most certainly differ depending on the level of animal-food restriction. A large cross-sectional study (*n* = 1475) of omnivores (*n* = 155), pesco-vegetarians (*n* = 145), semi-vegetarians (defined in this study as those who consume meat, fish, or poultry no more than once per week) (*n* = 498), vegetarian (*n* = 573), and vegans (*n* = 104) compared macro- and micronutrient intakes by dietary pattern [100]. Data indicated that omnivores consumed more total energy, saturated fat, cholesterol, sodium, and protein but less fiber, calcium, and iron than vegetarians [100]. Vegans consumed the least energy, saturated fat, sodium, and calcium, but the most fiber and iron. Other studies with smaller sample sizes have largely substantiated these observations [101,102,103]. In spite of the higher iron content in vegan diets, typically vegetarians have lower serum ferritin (iron stores) than meat-eaters, likely due to the reduced bioavailability of the type of iron found in plants. As high iron stores may be a risk factor for developing some chronic diseases, a fine balance must be achieved to optimize oxygen-carrying capacity of blood while avoiding increased risk of non-communicable diseases [104]. Conversely, low iron stores can result in iron-deficiency anemia, which can be problematic for athletic performance, particularly for endurance events, as iron plays a critical role in oxygen transport [105]. Given the key role of carbohydrate for energy production during exercise, particularly at high intensities, and of protein for building and repairing muscle tissue, differences in patterns of consumption between vegetarians and omnivores could theoretically lead to differences in exercise performance and recovery capacity. Not only are there documented specific nutrient intake differences between vegetarian and omnivores, but overall diet quality has also been assessed. Among a group of vegan, vegetarian, and omnivore recreational runners, overall diet quality scores were higher among vegans and vegetarians as compared to omnivores [106]. 

## 5. Exercise Performance and Plant-Based Diets

Athletes training for endurance sports versus strength and power-oriented sports have different training and fueling needs. As such, following a vegetarian diet may have different effects on performance outcomes for these athletes. Some studies have assessed the diet’s potential for impact on performance outcomes indirectly by measuring maximal oxygen uptake, strength, blood acid-base status, acute MPS, and chronic muscle growth instead of actual performance in sporting events, while others have measured performance outcomes such as time to exhaustion when cycling. The next sections will describe what is known currently about dietary pattern associations with outcomes related to endurance exercise and strength or power-focused exercise. 

A recent systematic review by Craddock and colleagues (2016) summarized much of the literature to date pertaining to a vegetarian diet and strength, anaerobic, and aerobic exercise performance. Given the search terms used, only eight studies were included in the review, highlighting the dearth of literature directly examining the impact of vegetarian diet upon performance. Furthermore, only three of these studies focused on strength training and power, four assessed both anaerobic and aerobic performance, and only one examined the impact of endurance exercise on immune markers. Of the studies reviewed (seven RCTs and one cross-sectional study), no differences in strength, anaerobic, or aerobic performance were identified, leading authors to conclude that vegetarian diets neither improved nor decreased performance [107]. These studies, in addition to other more recent additions to the literature, are described below. 

### 5.1. Endurance Exercise 

The only cross-sectional study reviewed by Craddock was by Hanne and colleagues (1986), comparing vegetarian and omnivore athletes (primarily endurance athletes) matched by type of sport on a number of parameters. Maximal oxygen uptake (VO2 max) was indirectly predicted by a stress test on a cycle ergometer, and a Wingate anaerobic test provided measurements of total power, peak power, and percent of fatigue. Neither aerobic nor anaerobic capacity significantly differed between vegetarians and omnivores [108]. While this study is one of the few to compare vegetarians who had adhered to their dietary pattern for at least 2 years, a drawback was that maximal oxygen uptake was only indirectly estimated. Additionally, there was no analysis of nutrient composition of the diet.

The intervention studies reviewed by Craddock included vegetarian interventions that lasted 4 days (Hietavala 2012), 5 weeks (Baguet 2011), 6 weeks (Richter 1991, Raben 1992), and 12 weeks (Campbell 1999, Wells 2003, Haub 2005). In a randomized cross-over design with a 16-day washout period, Hietavala and colleagues (2012) examined the impact of adopting a vegetarian diet for four days prior to completing a graded exercise test on a cycle ergometer compared to adhering to participants’ normal diets. Participants on the vegetarian diet had higher oxygen consumption at a given workload, although this did not reduce their maximal aerobic performance. However, not only was the source of the dietary protein changed for participants in this study, they also had their habitual daily protein intake of 1.59 g/kg reduced by nearly half to 0.8 g/kg. Thus, it was not possible to determine whether dietary protein amount, source, or another dietary change (such as the concurrent reduction in total calories and dietary fat) may have influenced these performance results [109]. 

As the ability to train well may be compromised during periods of sickness, having a strong immune system is important for athletes. A 6-week crossover design with a 4-week washout period comparing immune function among male athletes consuming either a lacto-ovo vegetarian (LOV) or mixed diet was conducted by Richter and colleagues (1991) and was included in Craddock’s review [110]. Macronutrient contribution to the diet was consistent between dietary patterns (57% carbohydrate, 14% protein, 29% fat). The meat-containing diet provided 69% of the protein from animal sources, whereas the vegetarian diet had 18% of the protein from animal sources. Training volume was similar during both diets, and maximal oxygen uptake did not change over time. A blood sample was collected 36 h after the last training period for both diet periods to determine the concentration of various mononuclear cells and the activity of natural killer cells. The number of CD3^+^, CD4^+^, CD8^+^, CD14^+^, and CD16^+^ cells were similar between groups, as was the activity of natural killer cells. 

Another cross-over design (Raben 1992) examined the impact of following a vegetarian diet for 6 weeks on a number of physiologic and performance outcomes among trained male endurance athletes (mean maximal oxygen uptake (VO2max) 67 mL/kg/min). Mixed diets included 58% of calories from carbohydrates, 27% by fat, and 15% by protein. Vegetarian diets similarly had 58% of energy from carbohydrates, 28% from fat, and 14% from protein. Additionally, the amino acid composition of the diet was not significantly different between groups. To assess exercise endurance, participants began a test on either a cycle ergometer or treadmill at a workload corresponding to 100-120 heart rate beats per minute (bpm). Every 15 min the workload increased corresponding to an additional 10-15 bpm. This was continued until exhaustion, and time to exhaustion was used as an indicator of exercise endurance capacity. Maximal voluntary contraction and isometric endurance at 35% of maximal voluntary contraction for elbow flexors and the quadriceps were determined by a strain-gauge when the participant was sitting upright. There were no significant differences between groups for VO2 max, time to exhaustion on the endurance test, maximal voluntary contraction, or isometric endurance upon adoption of the vegetarian diet [111]. 

The 12-week interventions reviewed by Craddock (Campbell 1999, Wells 2003, Haub 2005) all compared strength gains following a 12-week resistance training program between participants randomized to an LOV diet or to a meat-containing control diet. In each case, there were no significant differences between groups for strength on any test, except for knee extensions in Wells’ study (2012), in which the LOV group had greater increases in strength. While these studies did not directly measure sport-specific performance, strength development is a critical component for excelling in many high-level athletic endeavors and therefore relates to athletic success. One drawback, however, is that training status of the participants was unspecified in Wells’ and Haub’s studies. 

The only study assessing muscle buffering capacity, an important function for high-intensity activity, reviewed by Craddock et al. compared the effect of 5 weeks of sprint training coupled with adherence either to a vegetarian or mixed diet (Baguet 2011) [112]. Muscle carnosine content of the soleus, gastrocnemius lateralis, and tibialis anterior were measured by proton magnetic resonance spectroscopy (H-MRS), and non-bicarbonate muscle buffering capacity, carnosine content, and carnosine synthase mRNA expression were determined by true-cut biopsy of the gastrocnemius lateralis. Mean power output during six repeated 6-second sprints on a cycle ergometer was also measured pre- and post-dietary intervention. There was a significant time by group interaction for muscle carnosine content of the soleus in which the vegetarian group had a non-significant decrease and the mixed diet had a non-significant increase. However, in vitro muscle buffering capacity and peak power output did not differ between groups. 

Since the publication of Craddock’s review, several other studies have been published comparing vegetarian and omnivore athletes’ performance. Of these, one cross-sectional study compared maximal oxygen uptake (VO2 max), peak torque using an isokinetic dynamometer for knee extensions and flexions, and body composition between 27 vegetarian and 43 omnivore male and female endurance athletes. This study also included a 7-day food log to compare nutrient intake between groups. Results indicated that although total protein intake was lower among vegetarians, protein as a function of body weight (grams/kg) did not differ significantly by group (1.2 ± 0.3 and 1.4 ± 0.5 g per kg body mass for vegetarians and omnivores, respectively), and both groups were consuming protein within the range recommended for endurance athletes [85]. Peak torque when doing leg extensions and flexions did not differ significantly by diet group, and maximal oxygen uptake did not differ significantly for males (62.6 ± 15.4 and 55.7 ± 8.4 mL/kg/min respectively). However, female vegetarian athletes had higher VO2max values (53.0 ± 6.9 and 47.1 ± 8.6 mL/kg/min for vegetarians and omnivores, respectively) [113]. 

Blancquaret and colleagues (2018) conducted a three-arm, 6-month intervention in which women were randomized either to continuing an omnivorous diet, to adopting a vegetarian diet without supplementation, or to a vegetarian diet with daily β-alanine (0.8–0.4 g/day) and creatine (one gram creatine monohydrate) supplementation [114]. Supplementation status was double-blinded. Researchers measured muscle and plasma creatine, and carnitine and carnosine homeostasis at baseline, after 3 months, and after 6 months through a fasted venous blood sample, proton magnetic resonance spectroscopy (^1^H-MRS), and muscle biopsies. VO2 max and time to exhaustion on an incremental cycling test pre- and post-intervention were compared as well. Muscle total creatine declined only among the vegetarian plus placebo group after 3 months, whereas there was a main effect for time with no differences by group for carnitine moieties to decrease over time. Whereas fasting plasma β-alanine concentrations remained stable for the control and vegetarian plus placebo groups, the vegetarian plus supplement group increased plasma β-alanine concentration. This pattern was observed for soleus and gastrocnemius muscle β-alanine content as well. In spite of these differences, VO2max and time to exhaustion did not differ between groups at any time period. 

Additionally, recent case studies have also highlighted the capabilities of prominent vegan endurance athletes [115,116]. A case study by Wirnitzer and Kornexel (2017) reported energy intake and performance data for a female vegan cyclist competing in the 8-day Transalp Challenge 2004 (662 km, total altitude: 22,500 m). Given the grueling nature of the race, athletes must compete in teams of two for safety purposes. Prior to the race, the athlete trained about 25 h per week for 1 year. Relative peak power output during a pre-race laboratory assessment on an incremental cycling test was 4.6 watts per kg. Percent of peak power output and heart rate maximum were monitored continuously during the race. Over the course of the race, her body weight remained stable, indicating appropriate fueling and hydration practices, and she finished in 16th place out of 64 teams in the mixed category, meeting her goal of finishing in the top 20 [115]. 

Another case study by Leischik and Spelsberg (2014) compared a vegan male Triple-Ironman distance athlete with 10 non-vegetarian Triple-Ironman distance athletes on the basis of echocardiography, spiroergometry, and a blood test. Triple-Ironman race distance includes 11.4 km swimming, 540 km cycling, and 126 km running. The vegan athlete completed the race in 41 h 18 min. The athlete had been adhering to a raw vegan diet for 6 years; prior to this, he followed a vegan diet for 3 years. Before becoming vegan, he was vegetarian for 13 years. Given the nature of the study, it is not possible to make statistical comparisons between the vegan athlete and the average values of the 10 non-vegetarian athletes. However, their VO2max, oxygen uptake at ventilatory anaerobic threshold, and percent of VO2 max at ventilatory anaerobic threshold were similar. The vegan athlete had lower heart rates at rest, ventilatory anaerobic threshold, and maximal work. Left ventricular end diastolic diameter, end diastolic volume, and stroke volume also appeared higher in the vegan athlete. No dietary deficiencies or impaired health were indicated by bloodwork [116]. While such case reports are hardly sufficient for making recommendations about the adequacy of a vegetarian or vegan diet for endurance athletes, a large study (target *n* = 1500) is in progress comparing running performance among omnivore, vegetarian, and vegan runners, and results are forthcoming [117]. This study may serve as a model for other studies that could investigate the impact of dietary pattern on other types of sport, such as triathlon or cycling. 

### 5.2. Strength and Power Exercise 

Equally important is the impact of adoption of a plant-based diet on strength and power performance. Some of this research has involved examining differences in the physiological concentrations of creatine and carnosine, and how these differences may influence anaerobic activities. Beta-alanine is the rate-limiting precursor to forming carnosine, a key buffer in human skeletal muscle tissue, and beta-alanine is present in meat, fish, and poultry. As there is a positive correlation between buffering capacity and high-intensity exercise performance, it is important to determine whether the adoption of a vegetarian diet could impact muscle buffering capacity and consequently sprint or high-intensity performance. Baguet and colleagues (2011) conducted a 5-week intervention in which participants were randomized to either a lacto-ovo vegetarian group (supplemented with 1 g of creatine in order to compensate for a lower creatine content) or an omnivorous diet, and all participants engaged in sprint training 2–3 times per week. Power output increased during sprints on a cycle ergometer for both groups, with no significant between-group differences, and muscle carnosine concentrations did not change significantly from baseline in either group. There was a significant time-by-group interaction for carnosine concentration in the soleus with a non-significant decrease in the vegetarian group and a non-significant increase in the omnivorous group, but this did not affect buffering capacity [112]. 

Creatine levels represent another important physiological difference between vegetarians and omnivores that could affect strength and power in particular. Vegetarians generally present with lower total creatine levels. However, this difference appears to make them more receptive to creatine supplementation and its effects, as vegetarian participants receiving a creatine supplement exhibited greater increases in total creatine, phosphocreatine, lean body mass, and total work performed doing leg extensions and flexions on an isokinetic dynamometer compared to omnivores receiving creatine supplementation and undergoing the same training program [118]. There were no significant differences in total work output at baseline between groups, in spite of the lower total creatine levels. 

A study by Novakova (2016) compared body carnitine stores and physical performance between vegetarians and omnivores who were not specifically athletes. Vegetarians had lower plasma carnitine stores, but comparable skeletal muscle carnitine stores to omnivores. Additionally, skeletal muscle phosphocreatine, glycogen, lactate, and ATP levels were not significantly different between groups. Performance outcomes also did not differ by diet group. Maximal work performed and maximal oxygen uptake relative to body weight did not differ significantly, nor did respiratory exchange ratio, blood lactate, or other muscle metabolite levels during submaximal exercise [119].

Shomrat and colleagues (2000) also conducted a creatine supplementation study among vegetarians and omnivores, and compared peak and mean power output during modified Wingate tests, plasma creatine, and changes in lean body mass. At baseline, the only difference between vegetarians and omnivores was plasma creatine levels, a difference that did not significantly affect mean or peak power output during the modified Wingate tests. Post-supplementation, both groups had similar increases in mean power output, but only omnivores increased their peak power output [120]. 

### 5.3. Other Aspects

Increasingly, healthcare professionals and the public are recognizing the importance of quality of life as part of health and wellness. To compare quality of life scores between vegan, vegetarian, and omnivore male and female runners, Boldt and colleagues (2018) administered the World Health Organization Quality of Life Brief questionnaire to 281 recreational runners. This questionnaire addressed physical health, psychological wellbeing, social relationships, and one’s environment (e.g., satisfaction with where one lives and works, financial freedom, and access to healthcare), broadly representing overall life satisfaction. All diet groups reported high quality of life scores without significant differences between groups, leading authors to conclude that vegan and vegetarian diets represented viable options in support of high quality of life similar to meat-containing diets [121]. 

Exercise clearly is important for promoting optimal health, yet it also produces free radicals, compounds known to play a role in some conditions such as cardiovascular disease and the development of some cancers. Antioxidants found in fruits and vegetables help to neutralize free radicals. As such, it has been hypothesized that a plant-based diet higher in fruits and vegetables may help combat free radical production generated during exercise. To explore this assertion, Trapp and colleagues (2010) conducted a review of the literature on this topic. Although typically vegetarians and vegans consume more antioxidants such as vitamins C, E, and beta-carotene, this has not definitively translated to higher antioxidant status and reduced levels of oxidative stress in response to exercise. Most studies have simply examined nutrient intake and have not considered the effect on exercise-induced oxidative stress, which is a key area for future research [122]. 

## 6. Conclusions

In spite of differences in macro- and micronutrient intake between vegetarians and omnivores, as well as some physiological differences such as lower total body creatine and plasma carnitine among vegetarians, exercise performance does not appear to differ between dietary groups across multiple measures and types of activities. As much of this research has been done with recreational athletes; further work ought to be conducted among elite athletes to ascertain if differences may appear at higher levels of training and competition. 

## References

[1] Mintel Food and Drink Trends 2017. http://www.fpsa.org/wp-content/uploads/Global_Food_and_Drink_Trends_FSPA_March_17_2017.pdf.

[2] Mintel (2018). Global Food and Drink Trends 2018. https://gastronomiaycia.republica.com/wp-content/uploads/2017/10/informe_mintel_tendencias_2018.pdf.

[3] Catsburg C., Kim R.S., Kirsh V.A., Soskolne C.L., Kreiger N., Rohan T.E. (2015). Dietary patterns and breast cancer risk: A study in 2 cohorts. Am. J. Clin. Nutr..

[4] Tantamango-Bartley Y., Knutsen S.F., Knutsen R., Jacobsen B.K., Fan J., Beeson W.L., Sabate J., Hadley D., Jaceldo-Siegl K., Penniecook J. (2016). Are strict vegetarians protected against prostate cancer?. Am. J. Clin. Nutr..

[5] Orlich M.J., Singh P.N., Sabaté J., Jaceldo-Siegl K., Fan J., Knutsen S., Beeson W.L., Fraser G.E. (2013). Vegetarian dietary patterns and mortality in Adventist Health Study 2. JAMA Intern. Med..

[6] Orlich M.J., Singh P.N., Sabaté J., Fan J., Sveen L., Bennett H., Knutsen S.F., Beeson W.L., Jaceldo-Siegl K., Butler T.L. (2015). Vegetarian dietary patterns and the risk of colorectal cancers. JAMA Intern. Med..

[7] Orlich M.J., Fraser G.E. (2014). Vegetarian diets in the Adventist Health Study 2: A review of initial published findings. Am. J. Clin. Nutr..

[8] Kim M.-H., Choi M.-K., Sung C.-J. (2007). Bone mineral density of Korean postmenopausal women is similar between vegetarians and nonvegetarians. Nutr. Res..

[9] Kim M.-H., Bae Y.-J. (2015). Comparative study of serum leptin and insulin resistance levels between Korean postmenopausal vegetarian and non-vegetarian women. Clin. Nutr. Res..

[10] Yokoyama Y., Nishimura K., Barnard N.D., Takegami M., Watanabe M., Sekikawa A., Okamura T., Miyamoto Y. (2014). Vegetarian diets and blood pressure: A meta-analysis. JAMA Intern. Med..

[11] Quiles L., Portoles O., Sorlí J., Corella D. (2014). Short term effects on lipid profile and glycaemia of a low-fat vegetarian diet. Nutr. Hosp..

[12] Craig W.J. (2009). Health effects of vegan diets. Am. J. Clin. Nutr..

[13] Fraser G.E. (2009). Vegetarian diets: What do we know of their effects on common chronic diseases?. Am. J. Clin. Nutr..

[14] Bradbury K.E., Crowe F.L., Appleby P.N., Schmidt J.A., Travis R.C., Key T.J. (2014). Serum concentrations of cholesterol, apolipoprotein AI and apolipoprotein B in a total of 1694 meat-eaters, fish-eaters, vegetarians and vegans. Eur. J. Clin. Nutr..

[15] Chuang S.-Y., Chiu T.H., Lee C.-Y., Liu T.-T., Tsao C.K., Hsiung C.A., Chiu Y.F. (2016). Vegetarian diet reduces the risk of hypertension independent of abdominal obesity and inflammation: A. prospective study. J. Hypertens..

[16] De Biase S.G., Fernandes S.F.C., Gianini R.J., Duarte J.L.G. (2007). Vegetarian diet and cholesterol and triglycerides levels. Arq. Bras. Cardiol..

[17] Ferdowsian H.R., Barnard N.D. (2009). Effects of plant-based diets on plasma lipids. Am. J. Cardiol..

[18] Godos J., Bella F., Sciacca S., Galvano F., Grosso G. (2017). Vegetarianism and breast, colorectal and prostate cancer risk: An overview and meta-analysis of cohort studies. J. Hum. Nutr. Diet..

[19] Kahleova H., Tura A., Hill M., Holubkov R., Barnard N.D. (2018). A plant-based dietary intervention improves beta-cell function and insulin resistance in overweight adults: A 16-Week randomized clinical trial. Nutrients.

[20] Kahleova H., Pelikanova T. (2015). Vegetarian diets in the prevention and treatment of type 2 diabetes. J. Am. Coll. Nutr..

[21] Kahleova H., Levin S., Barnard N.D. (2018). Vegetarian dietary patterns and cardiovascular disease. Prog. Cardiovasc. Dis..

[22] Pettersen B.J., Anousheh R., Fan J., Jaceldo-Siegl K., Fraser G.E. (2012). Vegetarian diets and blood pressure among white subjects: Results from the Adventist Health Study-2 (AHS-2). Public Health Nutr..

[23] Szeto Y., Kwok T.C., Benzie I.F. (2004). Effects of a long-term vegetarian diet on biomarkers of antioxidant status and cardiovascular disease risk. Nutrition.

[24] Tonstad S., Stewart K., Oda K., Batech M., Herring R., Fraser G. (2013). Vegetarian diets and incidence of diabetes in the Adventist Health Study-2. Nutrition. Metab. Cardiovasc. Dis..

[25] Wang F., Zheng J., Yang B., Jiang J., Fu Y., Li D. (2015). Effects of vegetarian diets on blood lipids: A systematic review and meta-analysis of randomized controlled trials. J. Am. Heart Assoc..

[26] Metson G.S., Bennett E.M., Elser J.J. (2012). The role of diet in phosphorus demand. Environ. Res. Lett..

[27] Pimentel D., Pimentel M. (2003). Sustainability of meat-based and plant-based diets and the environment. Am. J. Clin. Nutr..

[28] Cordell D., Drangert J.-O., White S. (2009). The story of phosphorus: Global food security and food for thought. Global Environ. Chang..

[29] Baroni L., Cenci L., Tettamanti M., Berati M. (2007). Evaluating the environmental impact of various dietary patterns combined with different food production systems. Eur. J. Clin. Nutr..

[30] Leitzmann C. (2003). Nutrition ecology: The contribution of vegetarian diets. Am. J. Clin. Nutr..

[31] Leitzmann C. (2014). Vegetarian nutrition: Past, present, future. Am. J. Clin. Nutr..

[32] Marlow H.J., Hayes W.K., Soret S., Carter R.L., Schwab E.R., Sabate J. (2009). Diet and the environment: Does what you eat matter?. Am. J. Clin. Nutr..

[33] Reijnders L., Soret S. (2003). Quantification of the environmental impact of different dietary protein choices. Am. J. Clin. Nutr..

[34] Sabaté J., Soret S. (2014). Sustainability of plant-based diets: Back to the future. Am. J. Clin. Nutr..

[35] Sabaté J., Harwatt H., Soret S. (2016). Environmental nutrition: A new frontier for public health. Am. J. Public Health.

[36] Ruini L.F., Ciati R., Pratesi C.A., Marino M., Principato L., Vannuzzi E. (2015). Working toward healthy and sustainable diets: The “double pyramid model” developed by the Barilla Center for Food and Nutrition to Raise Awareness about the Environmental and Nutritional Impact of Foods. Front. Nutr..

[37] Soret S., Mejia A., Batech M., Jaceldo-Siegl K., Harwatt H., Sabaté J. (2014). Climate change mitigation and health effects of varied dietary patterns in real-life settings throughout North America. Am. J. Clin. Nutr..

[38] Springmann M., Godfray H.C.J., Rayner M., Scarborough P. (2016). Analysis and valuation of the health and climate change cobenefits of dietary change. Proc. Natl. Acad. Sci. USA.

[39] Stoll-Kleemann S., Schmidt U.J. (2017). Reducing meat consumption in developed and transition countries to counter climate change and biodiversity loss: A review of influence factors. Reg. Environ. Chang..

[40] Wickramasinghe K.K., Rayner M., Goldacre M., Townsend N., Scarborough P. (2016). Contribution of healthy and unhealthy primary school meals to greenhouse gas emissions in England: Linking nutritional data and greenhouse gas emission data of diets. Eur. J. Clin. Nutr..

[41] Kwok C.S., Umar S., Myint P.K., Mamas M.A., Loke Y.K. (2014). Vegetarian diet, Seventh Day Adventists and risk of cardiovascular mortality: A systematic review and meta-analysis. Int. J. Cardiol..

[42] Dinu M., Abbate R., Gensini G.F., Casini A., Sofi F. (2017). Vegetarian, vegan diets and multiple health outcomes: A systematic review with meta-analysis of observational studies. Crit. Rev. Food Sci. Nutr..

[43] Satija A., Bhupathiraju S.N., Rimm E.B., Spiegelman D., Chiuve S.E., Borgi L., Willett W.C., Manson J.E., Sun Q., Hu F.B. (2016). Plant-based dietary patterns and incidence of type 2 diabetes in US men and women: Results from three prospective cohort studies. PLoS Med..

[44] Turner-McGrievy G., Harris M. (2014). Key elements of plant-based diets associated with reduced risk of metabolic syndrome. Curr. Diabetes Rep..

[45] Sabaté J., Wien M. (2015). A perspective on vegetarian dietary patterns and risk of metabolic syndrome. Br. J. Nutr..

[46] Burkert N.T., Muckenhuber J., Großschädl F., Rásky E., Freidl W. (2014). Nutrition and health–the association between eating behavior and various health parameters: A matched sample study. PLoS ONE.

[47] Sutliffe J.T., Fuhrman J., Carnot M.J., Beetham R., Peddy M. (2016). Nutrient-dense, plant-rich dietary intervention effective at reducing cardiovascular risk factors for worksites: A pilot study. Altern. Ther. Health Med..

[48] Sutliffe J.T., Wilson L.D., de Heer H.D., Foster R.L., Carnot M.J. (2015). C-reactive protein response to a vegan lifestyle intervention. Complement. Ther. Med..

[49] Najjar R.S., Moore C.E., Montgomery B.D. (2018). A defined, plant-based diet utilized in an outpatient cardiovascular clinic effectively treats hypercholesterolemia and hypertension and reduces medications. Clin. Cardiol..

[50] Masset G., Vieux F., Verger E.O., Soler L.-G., Touazi D., Darmon N. (2014). Reducing energy intake and energy density for a sustainable diet: A study based on self-selected diets in French adults. Am. J. Clin. Nutr..

[51] Masset G., Soler L.-G., Vieux F., Darmon N. (2014). Identifying sustainable foods: The relationship between environmental impact, nutritional quality, and prices of foods representative of the French diet. J. Acad. Nutr. Diet..

[52] Carlsson-Kanyama A., Ekström M.P., Shanahan H. (2003). Food and life cycle energy inputs: Consequences of diet and ways to increase efficiency. Ecol. Econ..

[53] Machovina B., Feeley K.J., Ripple W.J. (2015). Biodiversity conservation: The key is reducing meat consumption. Sci. Total Environ..

[54] Smil V. (2000). Phosphorus in the environment: Natural flows and human interferences. Annu. Rev. Energy Env..

[55] Hallström E., Carlsson-Kanyama A., Börjesson P. (2015). Environmental impact of dietary change: A systematic review. J. Clean. Prod..

[56] Röös E., Karlsson H., Witthöft C., Sundberg C. (2015). Evaluating the sustainability of diets–combining environmental and nutritional aspects. Environ. Sci. Policy.

[57] Mejia A., Harwatt H., Jaceldo-Siegl K., Sranacharoenpong K., Soret S., Sabaté J. (2018). Greenhouse gas emissions generated by Tofu production: A case study. J. Hunger Environ. Nutr..

[58] Nijdam D., Rood T., Westhoek H. (2012). The price of protein: Review of land use and carbon footprints from life cycle assessments of animal food products and their substitutes. Food Policy.

[59] Stehfest E., Bouwman L., Van Vuuren D.P., Den Elzen M.G., Eickhout B., Kabat P. (2009). Climate benefits of changing diet. Clim. Chang..

[60] Wirsenius S., Azar C., Berndes G. (2010). How much land is needed for global food production under scenarios of dietary changes and livestock productivity increases in 2030?. Agric. Syst..

[61] McMichael A.J., Powles J.W., Butler C.D., Uauy R. (2007). Food, livestock production, energy, climate change, and health. Lancet.

[62] Churchward-Venne T.A., Pinckaers P.J., van Loon J.J., van Loon L.J. (2017). Consideration of insects as a source of dietary protein for human consumption. Nutr. Rev..

[63] van Huis A. (2016). Edible insects are the future?. Proc. Nutr. Soc..

[64] Halloran A., Roos N., Eilenberg J., Cerutti A., Bruun S. (2016). Life cycle assessment of edible insects for food protein: A review. Agron. Sustain. Dev..

[65] (2015). A Well-Fed World: Nourishing People sa. Feed: Meat Ratios. https://awfw.org/feed-ratios/.

[66] Amazon.com (2018). Search: Insect Protein. https://www.amazon.com/s/ref=nb_sb_noss_2?url=search-alias%3Daps&field-keywords=insect+protein+.

[67] Meyer N., Reguant-Closa A. (2017). “Eat as If You Could Save the Planet and Win!” Sustainability Integration into Nutrition for Exercise and Sport. Nutrients.

[68] Mamerow M.M., Mettler J.A., English K.L., Casperson S.L., Arentson-Lantz E., Sheffield-Moore M., Layman D.K., Paddon-Jones D. (2014). Dietary protein distribution positively influences 24-h muscle protein synthesis in healthy adults. J. Nutr..

[69] Nations U. Resolution adopted by the General Assembly on 20 December 2013 [on the report of the Second Committee (A/68/444)] 68/231. International Year of Pulses, 2016. Resolution. 2014. Contract No.: A/RES/68/231. http://www.un.org/en/ga/search/view_doc.asp?symbol=A/RES/68/231.

[70] Service USDoAER (2016). Food Availability and Consumption. https://www.ers.usda.gov/data-products/food-availability-per-capita-data-system/interactive-charts-and-highlights/.

[71] Daniel C.R., Cross A.J., Koebnick C., Sinha R. (2011). Trends in meat consumption in the USA. Public Health Nutr..

[72] van Vliet S., Burd N.A., van Loon L.J. (2015). The skeletal muscle anabolic response to plant-versus animal-based protein consumption. J. Nutr..

[73] Boirie Y., Dangin M., Gachon P., Vasson M.-P., Maubois J.-L., Beaufrère B. (1997). Slow and fast dietary proteins differently modulate postprandial protein accretion. Proc. Natl. Acad. Sci. USA.

[74] Glynn E.L., Fry C.S., Drummond M.J., Timmerman K.L., Dhanani S., Volpi E., Rasmussen B.B. (2010). Excess leucine intake enhances muscle anabolic signaling but not net protein anabolism in young men and women. J. Nutr..

[75] Tang J.E., Moore D.R., Kujbida G.W., Tarnopolsky M.A., Phillips S.M. (2009). Ingestion of whey hydrolysate, casein, or soy protein isolate: Effects on mixed muscle protein synthesis at rest and following resistance exercise in young men. J. Appl. Physiol..

[76] Devries M.C., McGlory C., Bolster D.R., Kamil A., Rahn M., Harkness L., Baker S.K., Phillips S.M. (2018). Leucine, not total protein, content of a supplement is the primary determinant of muscle protein anabolic responses in healthy older women. J. Nutr..

[77] Candow D.G., Burke N.C., Smith-Palmer T., Burke D.G. (2006). Effect of whey and soy protein supplementation combined with resistance training in young adults. Int. J. Sport Nutr. Exerc. Metab..

[78] Haub M.D., Wells A.M., Tarnopolsky M.A., Campbell W.W. (2002). Effect of protein source on resistive-training-induced changes in body composition and muscle size in older men. Am. J. Clin. Nutr..

[79] Reidy P.T., Borack M.S., Markofski M.M., Dickinson J.M., Deer R.R., Husaini S.H., Walker D.K., Igbinigie S., Robertson S.M., Cope M.B. (2016). Protein supplementation has minimal effects on muscle adaptations during resistance exercise training in young men: A double-blind randomized clinical trial. J. Nutr..

[80] Gilani G.S., Cockell K.A., Sepehr E. (2005). Effects of antinutritional factors on protein digestibility and amino acid availability in foods. J. AOAC Int..

[81] Friedman M., Brandon D.L. (2001). Nutritional and health benefits of soy proteins. J. Agric. Food Chem..

[82] Jansman A., Enting H., Verstegen M., Huisman J. (1994). Effect of condensed tannins in hulls of faba beans (Vicia faba L.) on the activities of trypsin (EC 2.4. 21.4) and chymotrypsin (EC 2.4. 21.1) in digesta collected from the small intestine of pigs. Br. J. Nutr..

[83] Bye J.W., Cowieson N.P., Cowieson A.J., Selle P.H., Falconer R.J. (2013). Dual effects of sodium phytate on the structural stability and solubility of proteins. J. Agric. Food Chem..

[84] Cheryan M., Rackis J.J. (1980). Phytic acid interactions in food systems. Crit. Rev. Food Sci. Nutr..

[85] Thomas D.T., Erdman K.A., Burke L.M. (2016). Position of the academy of nutrition and dietetics, dietitians of canada, and the american college of sports medicine: Nutrition and athletic performance. J. Acad. Nutr. Diet..

[86] Melina V., Craig W., Levin S. (2016). Position of the Academy of Nutrition and Dietetics: Vegetarian Diets. J. Acad. Nutr. Diet..

[87] Craig W.J., Mangels A.R. (2009). Position of the American Dietetic Association: Vegetarian diets. J. Am. Diet. Assoc..

[88] Association A.D. (2003). Position of the American Dietetic Association and Dietitians of Canada: Vegetarian diets. J. Acad. Nutr. Diet..

[89] (1989). Protein Quality Evaluation.

[90] (2013). Dietary Protein Quality Evaluation in Human Nutrition: Report of an FAO Expert Consultation.

[91] Hendriks W.H., van Baal J., Bosch G. (2012). Ileal and faecal protein digestibility measurement in humans and other non-ruminants–a comparative species view. Br. J. Nutr..

[92] Table M., Schlicker S., Yates A.A., Poos M. (2002). Food and Nutrition Board of the Institute of Medicine, The National Academies. Diet. Ref. Intakes Energy Carbohydr. Fiber Fat Fatty Acids Cholest. Protein Amino Acid..

[93] Otten J., Hellwig J., Meyers L. (2006). Dietary Reference Intakes. Institute of Medicine of the National Academies.

[94] Phillips S.M., Chevalier S., Leidy H.J. (2016). Protein “requirements” beyond the RDA: Implications for optimizing health 1. Appl. Physiol. Nutr. Metab..

[95] Moore D.R., Churchward-Venne T.A., Witard O., Breen L., Burd N.A., Tipton K.D., Phillips S.M. (2015). Protein ingestion to stimulate myofibrillar protein synthesis requires greater relative protein intakes in healthy older versus younger men. J. Gerontol. A Biol. Sci. Med. Sci..

[96] Phillips S.M., Van Loon L.J. (2011). Dietary protein for athletes: From requirements to optimum adaptation. J. Sports Sci..

[97] Doering T.M., Reaburn P.R., Phillips S.M., Jenkins D.G. (2016). Postexercise dietary protein strategies to maximize skeletal muscle repair and remodeling in masters endurance athletes: A review. Int. J. Sport Nutr. Exerc. Metab..

[98] Holwerda A.M., Kouw I.W., Trommelen J., Halson S.L., Wodzig W.K., Verdijk L.B., van Loon L.J. (2016). Physical activity performed in the evening increases the overnight muscle protein synthetic response to presleep protein ingestion in older men. J. Nutr..

[99] Trommelen J., van Loon L.J. (2016). Pre-Sleep protein ingestion to improve the skeletal muscle adaptive response to exercise training. Nutrients.

[100] Clarys P., Deliens T., Huybrechts I., Deriemaeker P., Vanaelst B., De Keyzer W., Hebbelinck M., Mullie P. (2014). Comparison of nutritional quality of the vegan, vegetarian, semi-vegetarian, pesco-vegetarian and omnivorous diet. Nutrients.

[101] Larsson C.L., Johansson G.K. (2002). Dietary intake and nutritional status of young vegans and omnivores in Sweden. Am. J. Clin. Nutr..

[102] Janelle K.C., Barr S.I. (1995). Nutrient intakes and eating behavior see of vegetarian and nonvegetarian women. J. Am. Diet. Assoc..

[103] Haddad E.H., Berk L.S., Kettering J.D., Hubbard R.W., Peters W.R. (1999). Dietary intake and biochemical, hematologic, and immune status of vegans compared with nonvegetarians. Am. J. Clin. Nutr..

[104] Haider L.M., Schwingshackl L., Hoffmann G., Ekmekcioglu C. (2018). The effect of vegetarian diets on iron status in adults: A systematic review and meta-analysis. Crit. Rev. Food Sci. Nutr..

[105] Alaunyte I., Stojceska V., Plunkett A. (2015). Iron and the female athlete: A review of dietary treatment methods for improving iron status and exercise performance. J. Int. Soc. Sports Nutr..

[106] Turner-McGrievy G.M., Moore W.J., Barr-Anderson D. (2016). The interconnectedness of diet choice and distance running: Results of the Research Understanding the Nutrition of Endurance Runners (RUNNER) Study. Int. J. Sport Nutr. Exerc. Metab..

[107] Craddock J.C., Probst Y.C., Peoples G.E. (2016). Vegetarian and omnivorous nutrition—Comparing physical performance. Int. J. Sport Nutr. Exerc. Metab..

[108] Hanne N., Dlin R., Nrotstein A. (1986). Physical fitness, anthropometric and metabolic parameters in vegetarian athletes. J. Sports Med. Phys. Fit..

[109] Hietavala E.-M., Puurtinen R., Kainulainen H., Mero A.A. (2012). Low-protein vegetarian diet does not have a short-term effect on blood acid–base status but raises oxygen consumption during submaximal cycling. J. Int. Soc. Sports Nutr..

[110] Richter E.A., Kiens B., Raben A., Tvede N., Pedersen B.K. (1991). Immune parameters in male atheletes after a lacto-ovo vegetarian diet and a mixed Western diet. Med. Sci. Sports Exerc..

[111] Raben A., Kiens B., Richter E.A., Rasmussen L.B., Svenstrup B., Micic S., Bennett P. (1992). Serum sex hormones and endurance performance after a lacto-ovo vegetarian and a mixed diet. Med. Sci. Sports Exerc..

[112] Baguet A., Everaert I., De Naeyer H., Reyngoudt H., Stegen S., Beeckman S., Bennett P. (2011). Effects of sprint training combined with vegetarian or mixed diet on muscle carnosine content and buffering capacity. Eur. J. Appl. Physiol..

[113] Lynch H.M., Wharton C.M., Johnston C.S. (2016). Cardiorespiratory fitness and peak torque differences between vegetarian and omnivore endurance athletes: A cross-sectional study. Nutrients.

[114] Blancquaert L., Baguet A., Bex T., Volkaert A., Everaert I., Delanghe J., Petrovic M., Vervaet C., De Henauw S., Constantin-Teodosiu D. (2018). Changing to a vegetarian diet reduces the body creatine pool in omnivorous women, but appears not to affect carnitine and carnosine homeostasis: A randomised trial. Br. J. Nutr..

[115] Wirnitzer K.C., Kornexl E. (2014). Energy and macronutrient intake of a female vegan cyclist during an 8-day mountain bike stage race. Proceedings.

[116] Leischik R., Spelsberg N. (2014). Vegan triple-ironman (raw vegetables/fruits). Case Rep. Cardiol..

[117] Wirnitzer K., Seyfart T., Leitzmann C., Keller M., Wirnitzer G., Lechleitner C., Rüst C.A., Rosemann T., Knechtle B. (2016). Prevalence in running events and running performance of endurance runners following a vegetarian or vegan diet compared to non-vegetarian endurance runners: The NURMI Study. SpringerPlus.

[118] Burke D.G., Chilibeck P.D., Parise G., Candow D.G., Mahoney D., Tarnopolsky M. (2003). Effect of creatine and weight training on muscle creatine and performance in vegetarians. Med. Sci. Sports Exerc..

[119] Novakova K., Kummer O., Bouitbir J., Stoffel S.D. (2016). Hoerler-Koerner, U.; Bodmer, M.; Roberts, P.; Urwyler, A.; Ehrsam, R.; Krähenbühl, S. Effect of l-carnitine supplementation on the body carnitine pool, skeletal muscle energy metabolism and physical performance in male vegetarians. Eur. J. Nutr..

[120] Shomrat A., Weinstein Y., Katz A. (2000). Effect of creatine feeding on maximal exercise performance in vegetarians. Eur. J. Appl. Physiol..

[121] Boldt P., Knechtle B., Nikolaidis P., Lechleitner C., Wirnitzer G., Leitzmann C., Rosemann T., Wirnitzer K. (2018). Quality of life of female and male vegetarian and vegan endurance runners compared to omnivores–results from the NURMI study (step 2). J. Int. Soc. Sports Nutr..

[122] Trapp D., Knez W., Sinclair W. (2010). Could a vegetarian diet reduce exercise-induced oxidative stress? A review of the literature. J. Sports Sci..

